# *Escherichia coli* Nissle 1917 engineered to express Tum-5 can restrain murine melanoma growth

**DOI:** 10.18632/oncotarget.20486

**Published:** 2017-08-24

**Authors:** Lian He, Huijun Yang, Fei Liu, Yiyan Chen, Sijia Tang, Wei Ji, Jianli Tang, Zhudong Liu, Yunjun Sun, Shengbiao Hu, Youming Zhang, Xiong Liu, Weitao Huang, Xuezhi Ding, Liqiu Xia

**Affiliations:** ^1^ Hunan Provincial Key Laboratory of Microbial Molecular Biology, State Key Laboratory of Developmental Biology of Freshwater Fish, College of Life Science, Hunan Normal University, Changsha 410081, Hunan, China

**Keywords:** Tum-5, E. coli Nissle 1917, lux operon, anti-angiogenesis, murine melanoma

## Abstract

Tumor growth and metastasis depend on angiogenesis. Thus, inhibiting tumor angiogenesis has become promising cancer therapeutic strategy in recent years. Tumstatin is a more powerful angiogenesis inhibitor than endostatin. Anti-angiogenic active fragment encoding amino acids 45–132 (Tum-5) of tumstatin was subcloned into four different inducible expression vectors and successfully solubly expressed in *Escherichia coli* BL21 (DE3) in this study. Subsequently, an anaerobic inducible expression vector was constructed under *Vitreoscilla* hemoglobin gene promoter *Pvhb* in *E. coli* Nissle 1917 (EcN). The secretory expression of Tum-5 in the engineered bacterium was determined *in vitro* and *in vivo* by Western blot or immunochemistry. The anti-tumor effect detection demonstrated that EcN could specifically colonize the tumor, and B16 melanoma tumor growth was remarkably restrained by EcN (Tum-5) in mice bearing B16 melanoma tumor. Abundant infiltrating inflammatory cells were observed in tumor areas of the EcN-treated group through hematoxylin and eosin staining, with a relatively reduced expression of endothelial marker platelet endothelial cell adhesion molecule-1 (PECAM-1/CD31) by immunofluorescence in tumor sections of EcN (Tum-5)-treated mice. No significant morphological differences were observed in the liver, kidney and spleen between EcN-treated mice and the control group, indicating that EcN was cleared by the immune system and did not cause systemic toxicity in mice. These findings demonstrated that the gene delivery of Tum-5 to solid tumors could be an effective strategy for cancer therapy.

## INTRODUCTION

The use of bacteria for cancer treatment has a long history [1–3]. The detailed mechanisms of their clinical effects are still unknown, but previous studies clearly showed that some bacteria could play important roles in treating some diseases, especially cancer. Over the last century, many genera of bacteria, including *Salmonella* [4, 5, 27], *Escherichia* [6, 7], *Clostridium* [8–10], *Bifidobacterium* [11–13] and *Listeria* [14, 15], have been shown to preferentially accumulate in hypoxic tumor areas. For nearly a decade, bacteria were used in cancer therapy by combining with chemotherapeutic drugs [38, 39] or delivering anti-tumor agents such as interfering RNAs [40, 42] prodrug activating enzymes [30] and cytokines [41, 43] into tumor tissues.

EcN was first isolated by the army surgeon Dr. Alfred Nissle in 1917 from the feces of a soldier who did not develop diarrhea during a severe outbreak of shigellosis [25]. Intestinal probiotics EcN have been licensed as a pharmaceutical in several countries for the treatment of diseases, such as diarrhea and colitis ulcerosa, affecting the digestive tract [16, 26]. Previous research revealed that EcN selectively colonized and replicated in the necrotic tumor tissue [7, 17, 28, 29]. Yunlei et al [29] used EcN as a delivery system for gene therapy on tumor-bearing mice. They sacrificed mice on 1, 3, 5, and 7 days after i.v. administration and cultured the lung, liver, spleen, kidney, heart, and tumor tissues. Then, the group found that EcN could only specifically target the tumor tissue. Jochen et al [17] injected EcN intravenously (i.v.), intraperitoneally (i.p.), or intratumorally (i.t.) into tumor-bearing mice to study its specific targeting property. They found that massive EcN colonized and replicated in tumors and no obvious difference was observed in the CFU/g isolated from organ tissues, regardless of inoculation route. The colonization of spleen and liver were significantly lower when EcN strains were used compared with *S. typhimurium*, and the non-pathogenic strains did not colonize the organs at all. Given its high tumor-selective replication and efficient clearance from the spleen and liver tissues, EcN was selected as a vector to specifically express Tum-5 in further studies.

Tumstatin, the NC1 domain fragment of the type IV collagen alpha3 chain, was deemed as a preferable tumor-specific angiogenesis inhibitor over endostatin. Tumstatin bound to avβ3 integrin and inhibited the growth of tumors in mouse models [18, 19]. The anti-angiogenic activity of tumstatin was located in the 54–132 amino acid region (Tum-5) through deletion mutagenesis [19]. This anti-angiogenic region was separate from the 185–203 amino acid region responsible for the anti-tumor activity. Tum-5 exerted the equal anti-angiogenesis effects similar to that induced by full-length tumstatin. This region bound predominantly to the β3 subunit of avβ3 integrin, and then inhibited the FAK, PI3K, and PKB cell signal transduction pathways, as well as reduced the phosphorylation of the mTOR kinase and eukaryotic initiation 4E. The latter effect led to the blocking of endothelial cell protein synthesis. As a result, the formation of new blood vessels were suppressed [20]. Finally, tumor growth, infiltration, and metastasis were suppressed after the up-regulation of Tum-5 expression.

In this study, the anti-tumor effect of EcN (Tum-5) on the mice bearing B16 mouse melanoma has been investigated *in vivo*. The engineered bacterium efficiently suppressed the growth of tumors by releasing Tum-5 protein and inhibiting tumor angiogenesis *in vivo*. This work laid an important foundation for bacteria-mediated tumor therapy.

## RESULTS

### Expression analysis of Tum-5 protein *in vitro*

**Figure 1:**
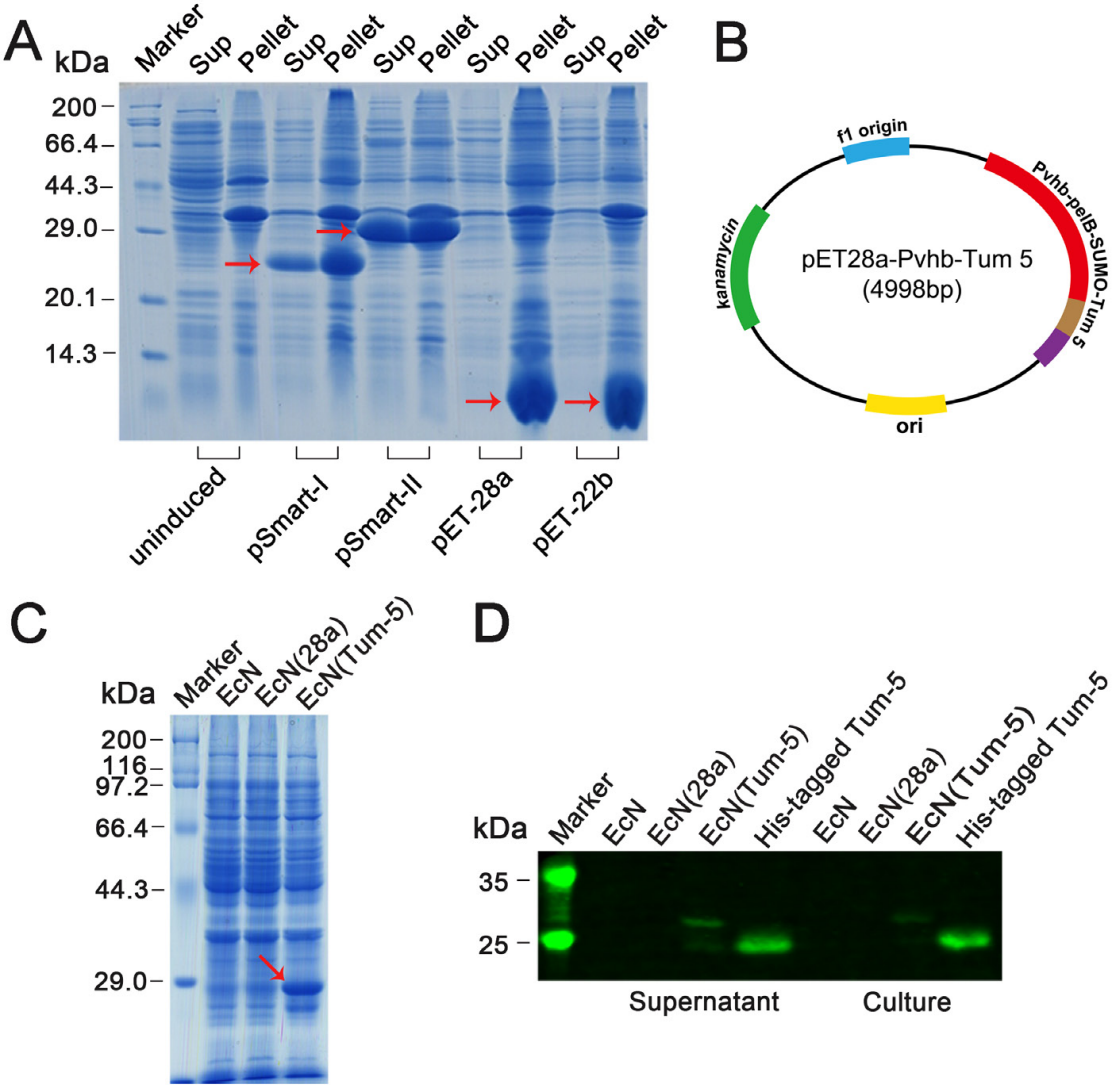
Characterization of Tum-5 expression *in vitro* **(A)** Coomassie Brilliant Blue staining of SDS–PAGE showing Tum-5 expression in *E.coli* BL21 (DE3). **(B)** Map of recombination plasmid containing *Tum-5* gene. **(C)** SDS–PAGE analysis of Tum-5 expression in EcN, EcN (28a), and EcN (Tum-5). **(D)** Western blot analysis of Tum-5 expression in cell lysates (supernatants) or culture supernatants of EcN, EcN (28a), and EcN (Tum-5).

To solubly express Tum-5 in prokaryotic expression system, the *Tum-5* gene was cloned into four inducible expression vectors with different labels (Supplementary Figure 1A and 1B). Western blot and mass spectrometry analysis demonstrated that the Tum-5 protein was successfully expressed in E. coli BL21 (DE3) (Supplementary Figure 1C and 1D). Coomassie Brilliant Blue staining analysis showed the recombinant proteins were souble expressed in the presence of SUMO and IF2 tag (Figure 1A). The continuous secretion of Tum-5 by the engineered bacterium is important in eliciting its anti-tumor activity. Therefore, Tum-5 was coexpressed with the pelB leader sequence under the oxygen-dependent promoter of the hemoglobin gene (*vhb*) of *Vitreoscilla* (Figure 1B and Supplementary Figure 2). The bacterial cells were cultivated in LB medium overnight and harvested by centrifugation. SDS–PAGE results confirmed that the Tum-5 protein was successfully expressed in EcN (Figure 1C). Western blot indicated that the Tum-5 protein was present in both cell lysate and medium supernatant of the EcN (Tum-5) (Figure 1D). The presence of two bands in Western blot may correspond to the presence of the pelB signal peptide before and after resection, because the gap between molecular sizes of these two bands is exactly equal to the size of pelB. The molecular weight of the SUMO tag was lower than that of the IF2 tag. Thus, the SUMO fusion system was selected to investigate the activity of the Tum-5 protein in the subsequent experiments.

### EcN specifically colonized and delivered Tum-5 to solid tumors

**Figure 2:**
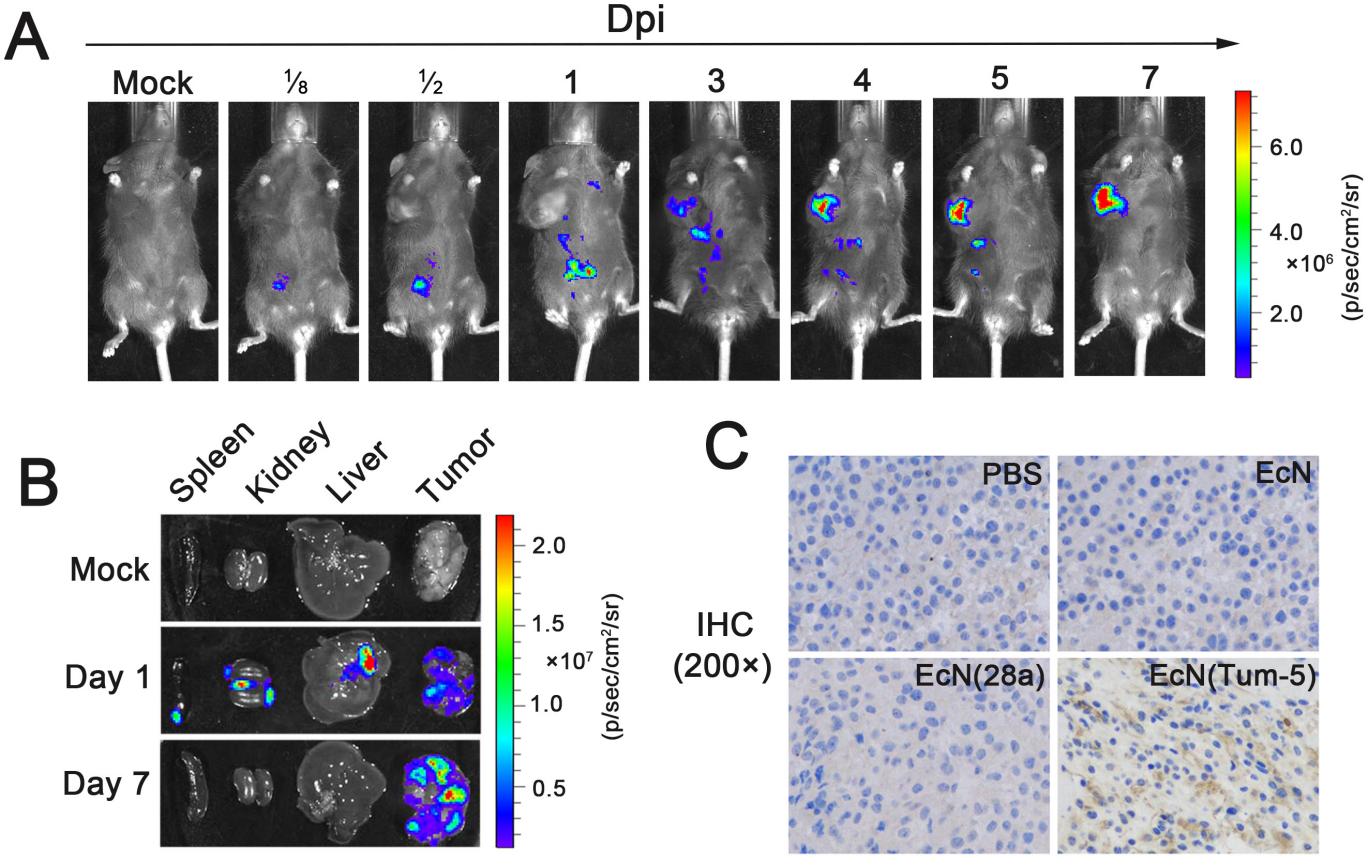
Colonization profile of EcN in the murine B16 tumor model and characterization of Tum-5 expression *in vivo* 1×10^5^ B16F10 cancer cells were inoculated into the right axillary subcutaneous of C57BL/6 mice. After 7–10 days, the mice were injected with 5×10^6^ CFU/100 μl EcN (Lux). **(A)** The distribution of the bacteria in the tumor-bearing mice were detected using IVIS. **(B)** The tumor, liver, kidney, and spleen of the mice were isolated at different times and observed using IVIS. **(C)** Tum-5 was confirmed to be successfully expressed in the tumor tissue by IHC (200×) using its specific antibody. Almost no positive signal was observed in tumor sections in the PBS-, EcN-, EcN (28a)-treated mice. “Dpi” means “days post infection.”

IVIS can be used to accurately observe the real-time distribution of bacteria in the animal body without any side effects [28, 35]. The colonization of EcN was investigated in mice bearing B16 melanoma at different time points after i.p. injecting 5×10^6^ CFU/100 μl EcN (Lux). The fluorescence signal was detected immediately at the injection site after intraperitoneal injection (Figure 2A). On the first day, the bacteria spread throughout all organs and tumors in the mice (Figure 2A and 2B). This result confirms that the animals have started to eliminate bacteria through their own immune system. Over the following 3–7 days, the bacteria steadily accumulated in tumors and were gradually removed from other organs. This finding implies that the bacteria require an adaptive process to specifically target the tumor (Figure 2A and 2B). Importantly, some mice possessed powerful immune systems and completely removed the bacteria from the liver, kidney, and spleen in 3–5 days without affecting the EcN-targeting tumors (data not shown). These discovery confirms that EcN exhibited excellent tumor-targeting properties.

To ensure that Tum-5 was successfully expressed in tumor areas to inhibit angiogenesis, we performed immunohistochemistry on each group of mouse tumors. The tumor-bearing mice were injected with sterilized PBS or 5×10^6^ CFU/100 μl bacteria. The tumor tissues were fixed in 4% paraformaldehyde and then embedded in paraffin after the mice were killed. Immunohistochemistry was used to determine whether Tum-5 could be expressed in tumor tissue. A significant gray signal was obviously displayed in the tumor section of the EcN (Tum-5)-treated group. However, the other groups did not display a positive signal (Figure 2C). The results demonstrated that Tum-5 was successfully expressed in the tumor region.

### EcN (Tum-5) significantly inhibited B16F10 melanoma proliferation in C57BL/6 mice

**Figure 3:**
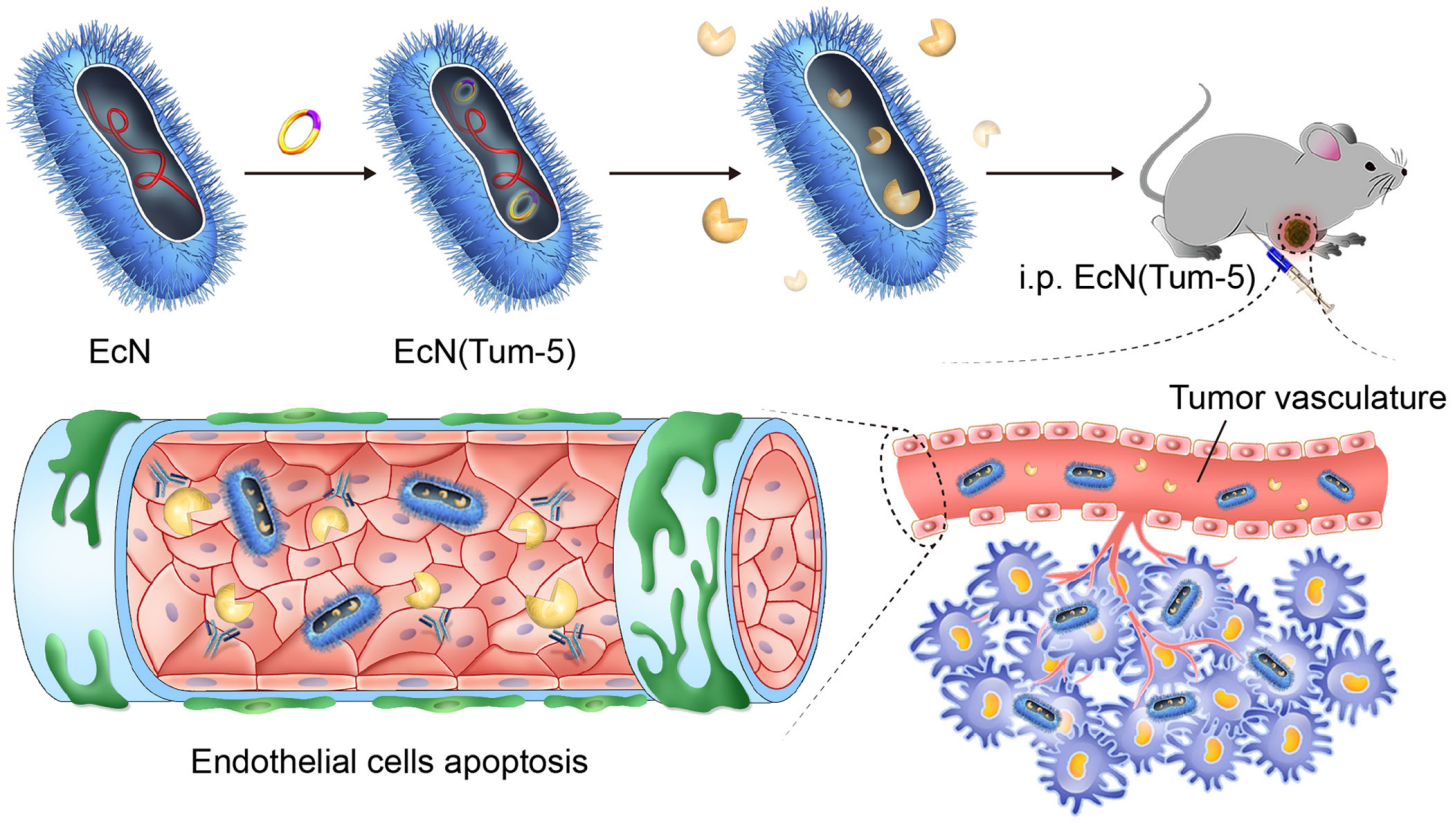
EcN (Tum-5) probiotics for cancer therapy Tum-5 protein (yellow) could be soluby expressed in EcN and secreted to the medium. The engineered bacteria (blue) were rapidly specially colonized in mouse tumors. Tum-5 bound to integrin receptors on the surface of vascular endothelial cells to induce endothelial cell apoptosis. This process would cause blood vessels to shrink, then the tumor growth was suppressed.

**Figure 4:**
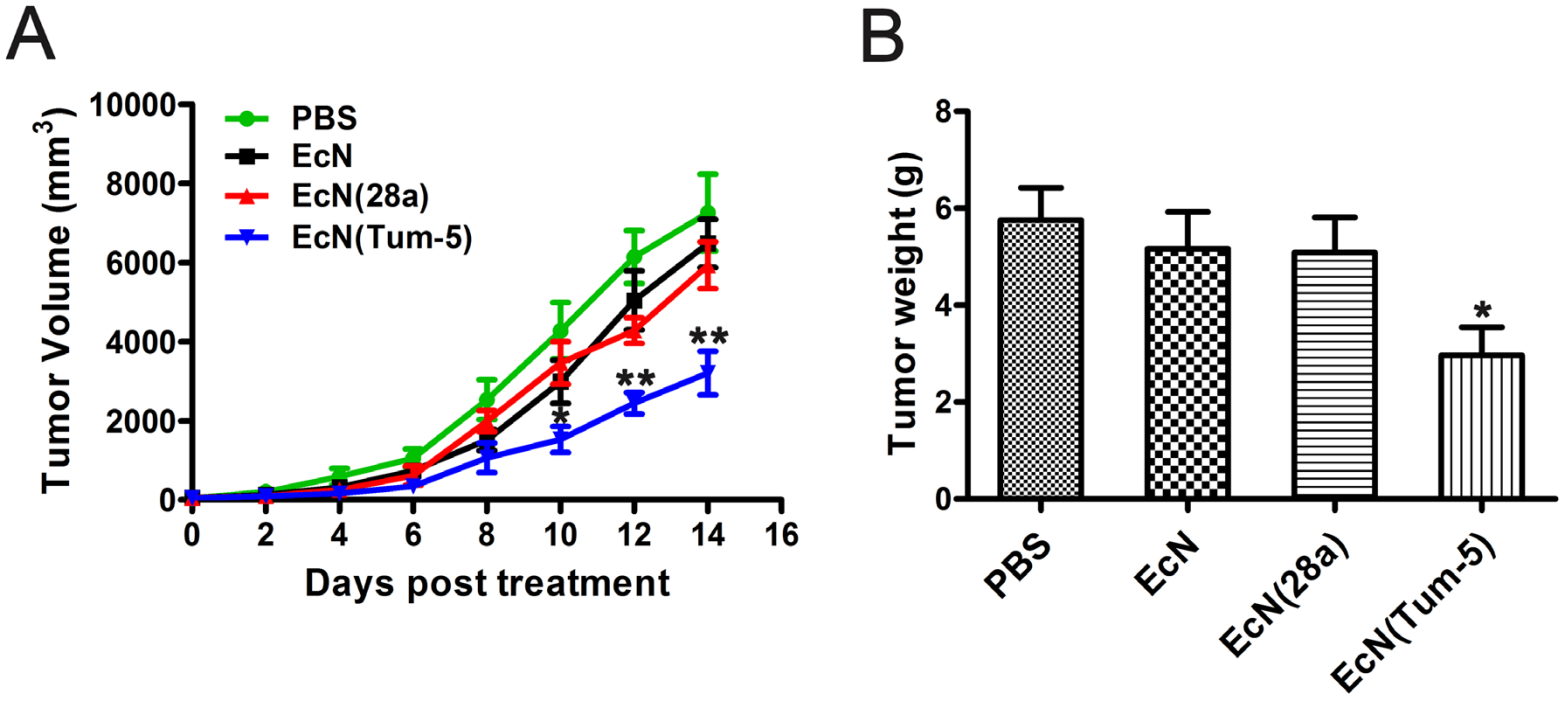
Inhibitory effect of EcN (Tum-5) on B16F10 melanoma tumor growth Therapeutic effect of EcN (Tum-5) on mice (n=6, 7, 8) bearing B16 melanoma tumors. Mice were treated by i.p. administration of either PBS or 5×10^6^ CFU/100 μl of EcN, EcN (28a), or EcN (Tum-5) at every 6-day intervals. Tumor volumes (mm^3^) were estimated using external calipers (values are expressed as means ± standard deviations [SDs]) **(A)**. B16 melanoma tumors were significantly inhibited by EcN (Tum-5) compared with the PBS control group. Mice were sacrificed, and tumor weights were measured **(B)**.

The *in vivo* anti-tumor activity of Tum-5 in xenograft mice with B16F10 melanoma cells was assessed (Figure 3). B16 melanoma cells were implanted into the right axillary subcutaneous area of the C57BL/6 mice. The resultant model was then used for investigating the anti-tumor effects of EcN (Tum-5). When the tumor volume reached approximately 60 mm^3^, the mice were randomly divided into four groups (n=6, 7, 8). Each group was injected with sterilized PBS or 5×10^6^ CFU/100 μl bacteria. The growth of the EcN (Tum-5)-treated tumors was remarkably suppressed. Compared with the mice in the PBS-, EcN-, and EcN (28a)-treated groups, the transplanted tumors of the EcN (Tum-5)-treated groups grew slower (Figure 4A). However, the groups that received EcN or EcN (28a) alone did not differ from the PBS group in terms of tumor suppression. The tumor weight of the EcN (Tum-5)-treated mice was significantly lower than those of the other groups (Figure 4B). The tumor volume of the PBS group reached up to 6813.66 ± 1021.38 mm^3^, but the tumor volume was significantly repressed by EcN (Tum-5), with a volume of 3205.83 ± 550.46 mm^3^. The tumor growth was inhibited by approximately 52.95% with respect to that of the PBS group (*P*<0.01) based on the formula (Table 1).

**Table 1:** The comparison of tumor volume, tumor weight of B16F10 cancer of the C57BL/6 mice

Group	Mean tumor volume (mm^3^)	Mean tumor weight (g)
PBS	6813.66 ± 1021.38	5.76 ± 1.15
EcN	5930 ± 591.14	5.17 ± 1.06
EcN (28a)	6481 ± 609.53	5.09 ± 1.02
EcN (Tum-5)	3205.83 ± 550.46 (52.95%)^**^	2.97 ± 1.15 (48.43%)^*^

### Histological morphology and MVD of the tumor tissues

**Figure 5:**
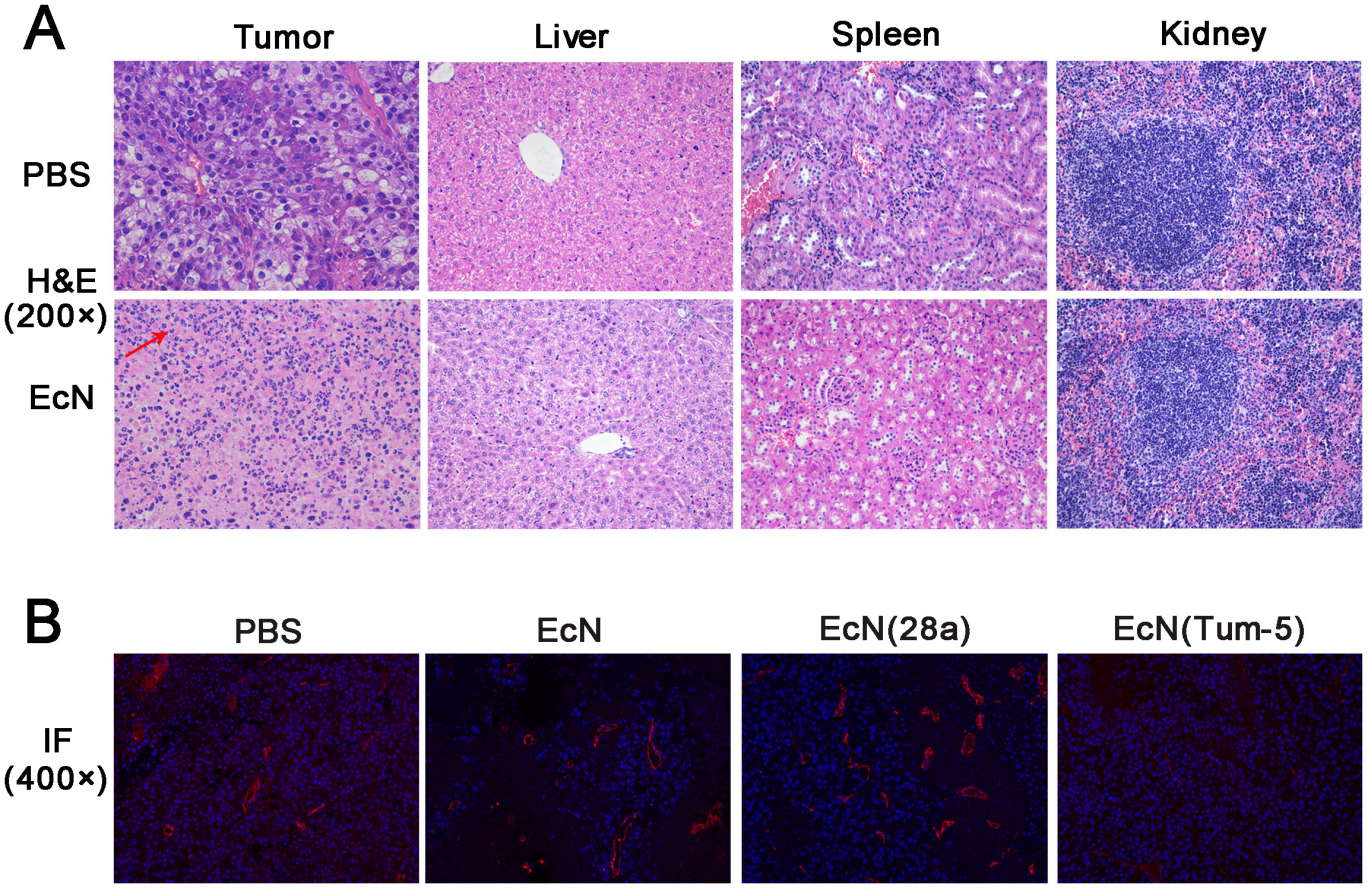
HE staining of tumor tissue sections and immunofluorescence of CD31 expression *in vivo* H&E staining (400×) of the tumor sections was used to observe biopsy pathological morphology **(A)**. IF staining (200×) for DAPI-stained nuclei (blue), CD31 (red) were evaluated by fluorescence microscopy **(B)**. Positive signals were hardly observed in the tumor sections of EcN (Tum-5)-treated mice.

Through H&E staining, the solid tumor sections generally showed enlarged tumor cells and nuclei. Nuclear chromatin was purple-blue under the hematoxylin stain, and the cytoplasm was stained red by eosin. Tumor tissue developed cell shrinkage and chromosome degradation when the cells were apoptotic [21, 22]. The morphological change of the tumor tissue in the tumor-burdened mice was observed after H&E staining. In the PBS-treated groups, tumor tissue exhibited a fairly complete structure and regular shapes. Moreover, no necrotic area was observed. However, massive infiltrating inflammatory cells (red arrow) were observed in the EcN-treated group (Figure 5A). No significant change in the histopathological morphology of organs were observed between the PBS and EcN groups throughout the experiment (Figure 5A). This result implies the negligible side effect on the liver, kidney, and spleen. The endothelial marker PECAM-1/CD31 is expressed in the endothelium of both lymphatic and blood vessels [23, 24]. Immunofluorescence assay revealed that the expression of CD31 and the microvessel density in the EcN (Tum-5)-treated group were significantly lower than those in the other three groups (Figure 5B). These findings illustrated that EcN (Tum-5) achieved an eminent anti-tumor activity, which might be due to the antiangiogenic effect of the Tum-5 protein. Therefore, the anti-angiogenesis effect of Tum-5 might suppress tumor angiogenesis and indirectly inhibit the growth, infiltration, and metastasis of tumors.

### Safety monitoring of *E. coli* Nissle 1917

**Figure 6:**
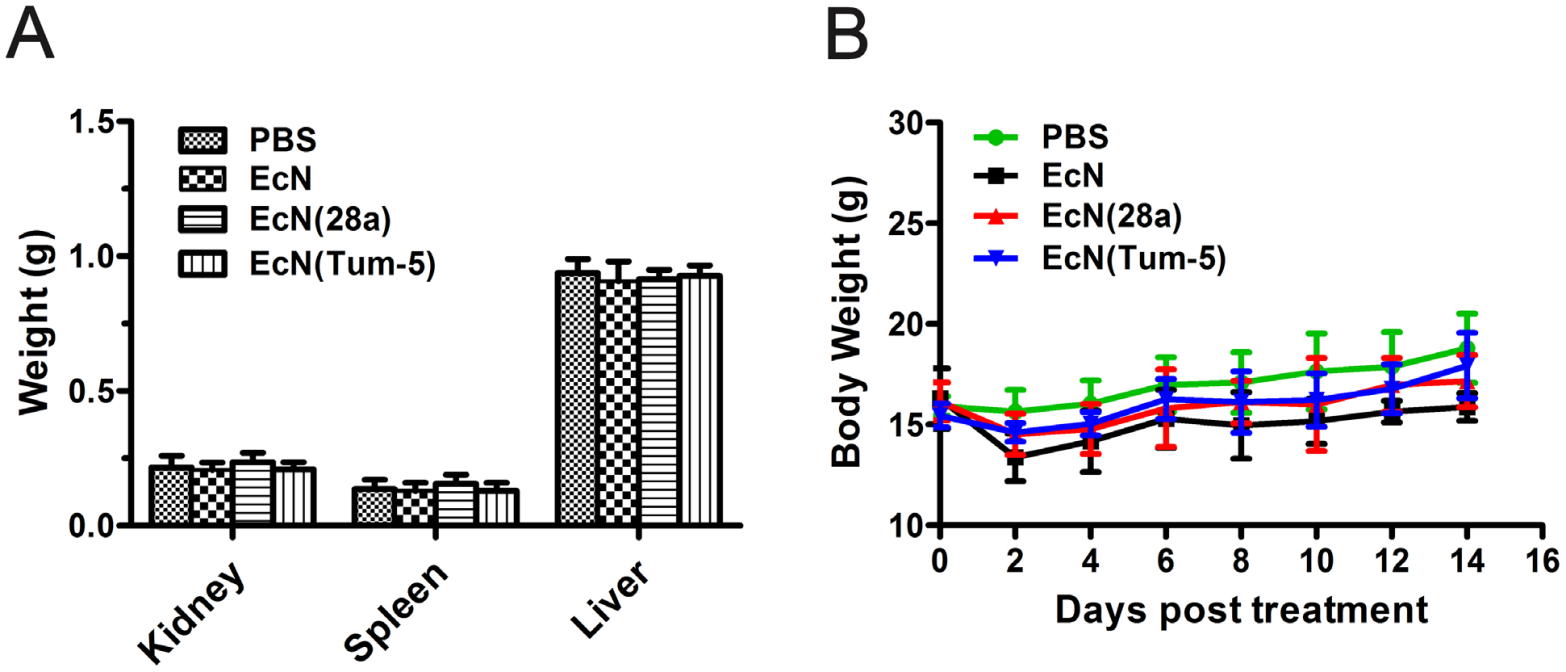
Body weight and kidney, spleen, and liver weight changes of mice before or after bacterial treatment Mice bearing B16 mouse melanoma were injected every 6 days with 5×10^6^ CFU/100 μl EcN, EcN (28a), EcN (Tum-5), or sterilized PBS three times. At the end of the experiment, the kidney, liver, and spleen were excised and weighed (means and SDs) **(A)**. The body weights were recorded at 2-day intervals. **(B)** No significant difference was observed between the PBS and EcN treatment groups.

EcN toxicity in tumor-bearing C57BL/6 mice (n=6, 7, 8) was evaluated every 6 days after 5×10^6^ CFU/100 μl EcN was administered. Body weight was measured every 2 days during treatment. At the end of the experiment, the liver, kidney, and spleen were excised and weighed. The organs of EcN-treated mice were not significantly differ from those of mice in PBS groups (Figure 6A). Although the body weight of the mice decreased slightly after injection, the weight immediately returned to normal in the next 2 days (Figure 6B). In general, these results demonstrated that the systemic administration of EcN did not substantially exert adverse effects on animals.

## DISCUSSION

In this study, the anti-tumor effect of EcN was assessed as a new drug carrier to deliver Tum-5 for cancer therapy. Tum-5 inhibited tumor growth, and this inhibitory effect was likely due to the transgenic expression of Tum-5 and the delivery of bacterial EcN, because the control groups failed to exhibit similar effects.

At present, drugs are administered to experimental animals through intravenous injection, intraperitoneal injection, and gavage. Intraperitoneal administration has its own unique advantages and has been successfully used in the treatment of multiple tumor models [45, 46, 47]. The peritoneum dense blood vessels and lymphatic vessels, thus allowing for the strong absorption capacity of solutes without overloading the heart. Solutes vacate the peritoneal cavity into the systemic circulation either by diffusion through the parietal/visceral peritoneum or by absorption through lymphatic tissue. The plasma-peritoneal barrier has unidirectional transport characteristics, with intraperitoneally administered substances appearing rapidly in systemic circulation, and with intravenously administered substances appearing slowly in peritoneal fluid. In a phase-I trial by Vasey et al [46], adenovirus dl1520 was intraperitoneally administered in patients with recurrent ovarian cancer. Tsutomu et al [47] intraperitoneally administered docetaxel to treat peritoneally disseminated cancer. Their results indicated that the intraperitoneal administration of docetaxel is likely an effective treatment method for peritoneally disseminated cancer. In addition, this method is not associated with increase systemic toxicity. Furthermore, given that intraperitoneally injected bacteria could migrate to regional lymph nodes, mesentery, and other tissues, the anti-angiogenic Tum-5 protein might be able to decrease tumor metastases to the lymph nodes and other organs.

The bacterial colonization of tumors was initially attributed to the hypoxic nature of solid tumors. The involvement of bacterial chemotaxis toward chemo-attractant compounds (e.g., aspartate, serine, citrate, ribose, or galactose) in necrotic regions of quiescent cancer cells has also been suggested as a contributing factor [44]. In this study, the mice were dissected to isolate the tumor, liver, kidney, and spleen. Then, the IVIS system was used to observe the distribution of bacteria in these organs after intraperitoneal injection. Although the bacteria were systemically distributed within a short period of time, the bacteria in other organs were removed by the immune system over time. Thus, EcN achieved an excellent targeting property to the anaerobic environment of tumor tissues, and the present experimental results also confirmed this view. These findings suggest that the intraperitoneal injection of bacteria also allowed the bacteria to specifically colonize in the tumors of the tumor-bearing mice. In addition, immunohistochemistry confirmed that Tum-5 was efficiently and consecutively expressed in the tumor tissues. These results confirmed that EcN could colonize the solid tumor areas. These findings thus lay an important foundation for future studies on other tumor-targeting bacteria.

As the anti-angiogenic active region of tumstatin, Tum-5 has been studied since its discovery [18, 19, 31, 32]. Li et al [33] placed Tum-5 in the pLXSN retroviral vector and found that the pLXSN-Tum-5 significantly inhibited the growth of human umbilical vein endothelial cells and H22 HCC cell tumor. Yanjie et al [34] confirmed that Tum-5 could inhibit the growth of S180 tumors in tumor-bearing mice. In the present study, the anti-angiogenic effects of Tum-5 and the anti-tumor effects exerted by EcN (Tum-5) *in vivo* were investigated using B16 melanoma tumor cells. The tumor volume and weight were significantly repressed by EcN (Tum-5). Notably, the tumor growth was extremely restrained by EcN (Tum-5), which led to 52.95% (*P*<0.01) and 48.43% (*P*<0.05) repression on tumor volume and tumor weight, respectively. The present research indicated that EcN (Tum-5) significantly inhibited the growth of B16 melanoma, whereas the body and tissue weight of mice were not affected.

Tumor angiogenesis is a critical feature of tumor growth, and some anti-angiogenesis agents were developed to cure tumor by blocking tumor angiogenesis [36, 37]. Immunofluorescence analysis with CD31 antibody indicated that the average microvessel density in the EcN (Tum-5) group was significantly lower than that in the PBS and EcN groups. These results suggested that Tum-5 exerted its anti-tumor activity by suppressing vascular endothelial cells. Whether EcN (Tum-5) can inhibit the angiogenesis of other tumor cells in tumor-bearing mice must be explored. Combining EcN (Tum-5) with radiotherapy, chemotherapy, or other cancer treatments also merits further study.

Interestingly, the body weight of each mouse group decreased slightly after injection but immediately returned to normal in the next 2 days for the entire treatment experiment. This result maybe the normal response of the mice to treatment, because the mouse weights in each group were stable throughout the treatment courses [7, 21]. In general, the PBS-treated mice held a slight advantage in body weight. Although the tumor volume was increasing, the mice became increasingly thin due to aggravating illness. As a result, the mouse weight did not show a significant upward trend. The mice in the EcN (Tum-5)-treated group exhibited improved physical fitness after the tumor volume was reduced by the therapeutic effect of Tum-5. Accordingly, no significant difference in weight was observed between the EcN (Tum-5)- and PBS-treated mice. The mice in the EcN group and the EcN (28a) group were similar to those in the PBS group. Therefore, no significant difference in body weight was observed among the four groups, and the results were reasonable.

In summary, bacteria engineered for Tum-5 gene delivery was successfully constructed. These bacteria were designed to specifically deliver gene to the tumor area and exert the gene’s anti-angiogenesis efficacy. Using tumor-targeting bacteria EcN to deliver therapeutic agents to solid tumors could serve as a prospective approach in future tumor therapy.

## MATERIALS AND METHODS

### Animal and cell culture

All animal experiments followed the National Institutes of Health Guide for the Care and Use of Laboratory Animals and were approved by the Animal Ethics Committee of Hunan Normal University. C57BL/6 mice (6–8 weeks) were fed under specific pathogen-free (SPF) conditions. B16F10 melanoma cells were cultured at 37 °C and in 5% CO_2_ atmosphere in 1640 supplemented with 10% heat-inactivated fetal bovine serum containing 100 U/ml penicillin and 100 μg/ml streptomycin.

### Gene cloning and soluble expression of Tum-5

DNA sequences of Tum-5 (amino acids 45–132 of tumstatin) were obtained by spliced overlap extension PCR technology. The primers used in the study were listed in Supplementary Table 1. The Tum-5 gene was inserted into the *Nco* I and *Xho* I restriction enzyme sites of pET-28a and pET-22b. *Tum-5* was cloned into *Bam*H I and *Xho* I restriction sites of pSmart-I (small ubiquitin-related modifier-SUMO fusion expression system) and pSmart-II (initiation factor-IF2 protein structure domain I fusion expression system). The resulting vectors were named pET-28a-Tum 5, pET-22b-Tum 5, pSmart-I-Tum 5, and pSmart-II-Tum 5. The four plasmids were transformed into *E. coli* BL21(DE3) and cultured at 37 °C in LB medium supplemented with 100 μg/ml ampicillin or 50 μg/ml kanamycin. Until the OD_600_ value reached 0.4–0.6, IPTG was added at a final concentration of 0.3 mM to induce Tum-5 protein expression at 30 °C. Cells were harvested by centrifugation at 8000 rpm after 4 h induction. After washed twice and resuspended in a mixture of 50 mM NaH_2_PO_4_ and 300 mM NaCl at pH 8.0, the cells were lysed by sonication, and the supernatants and pellets were analyzed by sodium dodecyl sulfate–polyacrylamide gel electrophoresis (SDS–PAGE) after centrifugation. The recombinant protein was cut with a surgical blade and identified by LTQ XL mass spectrometry (Thermo Fisher) after proteolysis.

### Construction of EcN expression strains

The *Vitreoscilla* hemoglobin gene promoter *Pvhb* was amplified from pET-28a-Pvhb-pelB-asp (Lab store). Meanwhile, the *Sumo*–*Tum 5* fragment was amplified from pSmart-I-Tum 5 (constructed in this study). Pvhb-pelB-SUMO-Tum 5 was obtained by overlap extension PCR and inserted into pET-28a after digestion by *Apa* I and *Xho* I. The sequenced vector was transformed into EcN by electroporation and named EcN (Tum-5). pET-28a was also transformed into EcN as negative control and named EcN (28a). EcN (Tum-5), EcN (28a) and EcN were cultured in LB medium for 10 h. Then, Western blot was used to confirm the expression of Tum-5 in both cell supernatant and culture supernatant by using Anti-6×His rabbit polyclonal antibody. The medium supernatant was precipitated with acetone containing 10% TCA and then washed with 90% acetone. Total cell protein (10 μg) was separated through SDS*–*PAGE and transferred to polyvinylidene difluoride membranes (Millipore) using a semidry method. The result was detected by IRDye® 680 conjugated goat anti-rabbit IgG (LI-COR, America) and observed by Odyssey Infrared Imaging System (LI-COR, America).

### Animal experiments

C57BL/6 female mice (6*–*8 weeks old) were purchased from the SLRC Laboratory Animal Company (Hunan, China). The animals were bred and maintained under SPF conditions for at least 3 days before use. For the melanoma tumor model, 1×10^5^ B16F10 cancer cells suspended in 100 μl PBS were injected into the right axillary subcutaneous of the C57BL/6 mice.

After the tumor volume reached 60 mm^3^, the mice were divided into four groups (6*–*8 mice per group) randomly. The mice were intraperitoneally (i.p.) injected with sterilized PBS, EcN, EcN (28a) or EcN (Tum-5) every 6 days at three times. The amount of bacteria injected was 5×10^6^ colony-forming units (CFU)/100 μl. The body weights and tumor volumes were measured every 2 days over the whole experiment. Then, all mice were sacrificed for analysis at day 14, and the weights of the tumor, liver, kidney, and spleen were measured at the end of treatment. The anti-tumor activities of the treatments were evaluated by tumor growth inhibition. Tumor volume (TV) was calculated according to the formula: TV (mm^3^) = d^2^ x D/2, where d and D are the shortest and the longest diameter, respectively. Tumor suppression percentage was calculated by the following computational formula: (control group − treatment group) / control group ×100 % (with tumor volume or tumor weight to calculate).

### Non-invasive *in vivo* imaging

To monitor bacteria distribution in mice after injection, a vector for the constitutive expression of *lux* was constructed by inserting the *lux*CDABE operon from *Photorhabdus luminescens* into the pET-28a vector. Then, the pET-28a-Lux plasmid was transformed into EcN by electroporation. When the tumor volume has grown to the appropriate size, the mouse was i.p. injected with 5×10^6^ CFU/100 μl EcN (Lux) to observe the colonization of the bacteria in the living body at different time points using *in vivo* imaging system (IVIS, Calipers). The mice were anesthetized with 2% isoflurane by using a XGI-8 gas system (Calipers).

### Histological morphology and microvessel density (MVD) of tumor

Upon isolation, tumors were fixed in 4% paraformaldehyde overnight after the mice were sacrificed and embedded in paraffin. Then, the tumors were prepared for hematoxylin and eosin (H&E) staining and immunofluorescent (IF) assay in accordance with standard laboratory procedures. DAPI was used to label cell nucleus and endothelial marker CD31 antibody was adopted to mark the microvessels. The specimens were observed and photographed under the microscope or fluorescence microscope. The expression of Tum-5 was assayed by immunohistochemical (IHC) staining using similar approaches. The liver, spleen, and kidney of the PBS group and EcN groups were also prepared for H&E staining to determine whether EcN has noticeable toxicity on the animals.

### Statistical analysis

All data were expressed as the mean ± standard deviation and analyzed using software IBM SPSS statistics 21.0. The statistical significance for all experimental groups was determined by student’s *t*-test. A difference with a *P* value of less than 0.05 was considered statistically significant, whereas *P* <0.01 was considered extremely significant.

## SUPPLEMENTARY MATERIALS FIGURES, TABLE



## Abbreviations

EcN: *Escherichia coli* Nissle 1917
PECAM-1/CD31: endothelial marker platelet endothelial cell adhesion molecule-1
H&E: hematoxylin and eosin staining
IF: immunofluorescent
IHC: immunohistochemical
IF: initiation factor
i.v.: intravenously
i.p.: intraperitoneally
i.t.: intratumorally
SDS–PAGE: sodium dodecyl sulfate–polyacrylamide gel electrophoresis
SUMO: small ubiquitin-related modifier
