# Supplementary material for: Escherichia coli Nissle 1917 engineered to express Tum-5 can restrain murine melanoma growth
Source: Oncotarget. 2017 Aug 24;8(49):85772–82. doi: 10.18632/oncotarget.20486 (PMC5689645; doi:10.18632/oncotarget.20486)
Supplement: Supplementary file 1 [file oncotarget-08-85772-s001.pdf]

Escherichia coli Nissle 1917 engineered to express Tum-5 can restrain murine melanoma growth

SUPPLEMENTARY MATERIALS

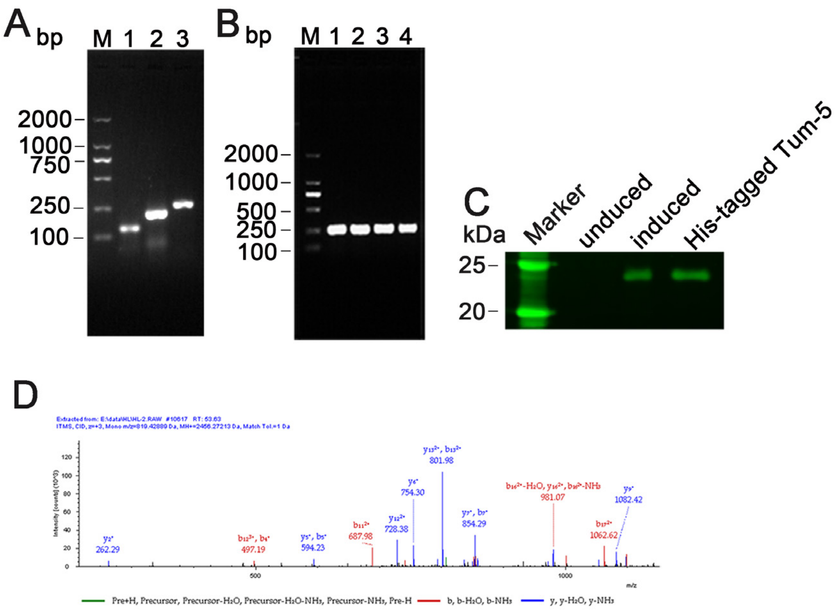

Supplementary Figure 1: Construction of Tum-5 induced expression vector and identification of recombinant protein. **(A)** Agarose gel electrophoresis of Tum-5 gene by splice PCR. M, DL2000 DNA Marker; lanes 1 to 3, products of three times splice PCR. **(B)** Identification of the *Tum-5* gene by PCR. M, DL2000 DNA Marker; lanes 1 to 4, PCR products amplified from pET-28a-Tum 5, pET-22b-Tum 5, pSmar-I-Tum 5, and pSmart-II-Tum 5 plasmids extracted from *E. coli* GB2005. **(C)** Western blot analysis of Tum-5 expression in *E. coli* BL21 (DE3). **(D)** Peptide mass fingerprinting of Tum-5 protein.

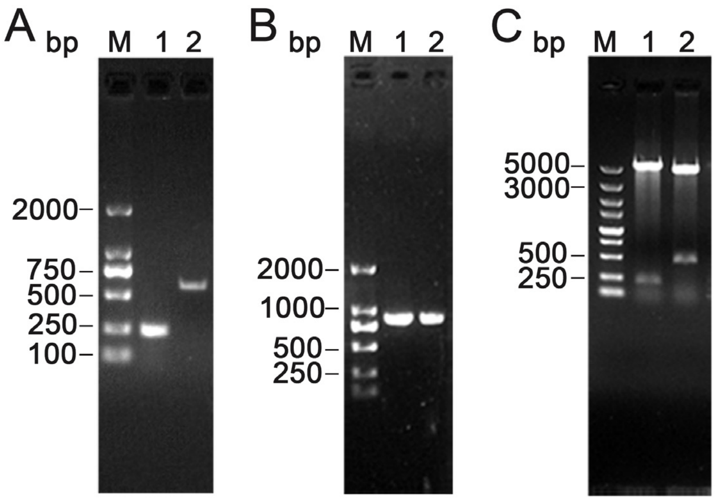

Supplementary Figure 2: Construction of Tum-5 hypoxia expression. **(A)** Agarose gel electrophoresis of *Tum-5* gene and *vhb* promoter. M, DL2000 DNA marker; lane 1, products of *vhb*; lane 2, products of *SUMO–Tum 5*. **(B)** Agarose gel electrophoresis of target fragments by overlapping PCR. M, DL 2000 DNA marker; lanes 1 and 2, products of full *Tum-5* nucleotide sequence. **(C)** Restriction digestion analysis of pET-28a (lane 1) and pET-28a-Pvhb-pelB-Tum 5 (lane 2).

Supplementary Table 1: Primers used in PCR amplification

Primers	DNA sequences (5′→3′)	PCR products
Tum 5-M	TGATGTATGTAATTTTGCATCTCGAAA<br>TGATTATTCATACTGGCTGTCAACACCAGCTC	
Tum 5-A-F	GCGATTTACCACAATGCCATTCTTATTCTGC<br>AATGTCAATGATGTATGTAATTTTGCAT	
Tum 5-A-R	AGGGCTCTGCCAGTAATGGGAGC<br>CATGTTCATTGGCATCAGAGCTGGTGTTGACAGCCA	
Tum 5-B-F	CCACGGACAAGACCTTGGAACTCTTG<br>GCAGCTGCCTGCAGCGATTTACCACAATGCCAT	282bp
Tum 5-B-R	ACCTTCACAAACAGTGCATCTGCTTA<br>TATAAGGCTCAAGGGCTCTGCCAGTAATGGGAG	
Tum 5-C-F-*Nco* I	CATGCCATGGGCAATCAAC<br>GAGCCCACGGACAAGACCTTGGAAC	
Tum 5-C-R-*Xho* I	CCGCTCGAGGGCGATCGCAGGACCTTCACAAACAGTGCATC	
Tum 5-F-*Nco* I	CATGCCATGGGGTTTTCTTTTCTTTT<br>TGTACAAGGAAATCAACGAGCCCACGGACA	
Tum 5-F-*Bam*H I	CGCGGATCCGGGTTTTCTTTTCTTTTTGT	282bp
Tum 5-R-*Xho* I	CCGCTCGAGGGCGATCGCAGGACCTTCAC	
vhb-F-*Apa* I	TGGGGGCCCTAGCTTACAGGACGCTGGGG	
vhb-R-SUMO	GAGTCCGACATATGGTGATGGTGAT<br>GGTGACCCATAGCCATCGCTGGTTGGGCAG	817bp
SUMO-F	ATGGGTCACCATCACCATCACC	
